# Development of Indirect Competitive ELISA for Lithospermic Acid B of *Salvia miltiorrhiza* with Its Specific Antibodies Generated via Artificial Oil Bodies

**DOI:** 10.3390/molecules24101952

**Published:** 2019-05-21

**Authors:** Yu-En Shih, Chao-Hsiang Chen, Nan-Hei Lin, Jason T.C. Tzen

**Affiliations:** 1 Graduate Institute of Biotechnology, National Chung-Hsing University, Taichung 402, Taiwan; ken23710@gmail.com; 2 Ko Da Pharmaceutical Co. Ltd., Taoyuan 324, Taiwan; gm@koda.com.tw; 3 Graduate Institute of Pharmacognosy, Taipei Medical University, Taipei 110, Taiwan; 4 Department of Biotechnology and Pharmaceutical Technology, Yuanpei University of Medical Technology, Hsinchu 300, Taiwan

**Keywords:** artificial oil bodies, caleosin, indirection competition ELISA, lithospermic acid B, *Salvia miltiorrhiza*

## Abstract

Lithospermic acid B (LSB), the major water-soluble ingredient of *Salvia miltiorrhiza* (Danshen), has been shown to be an active ingredient responsible for the therapeutic effects of this traditional Chinese herb used to treat cardiac disorders. This study aimed to develop an indirect competitive enzyme linked immunosorbent assay (ELISA) for the detection of LSB. Firstly, LSB was chemically conjugated to a modified oil-body protein, lysine-enriched caleosin, recombinantly expressed in *Escherichia coli*. Antibodies against LSB (Ab-LSB) were successfully generated by immunizing hens with artificial oil bodies constituted with the LSB-conjugated caleosin. Western blotting showed that Ab-LSB specifically recognized LSB, but not the carrier protein, lysine-enriched caleosin. To detect LSB via indirect competitive ELISA, LSB was conjugated with bovine serum albumin (LSB-BSA) and coated on a microplate. The binding between Ab-LSB and LSB-BSA on the microplate was competed dose-dependently in the presence of free LSB with a concentration ranging from 5 to 5 × 10^4^ ng/mL. The IC_50_ value was approximately determined to be 120 ng/mL for LSB regardless of its complex with a metal ion of Na^+^, K^+^ or Mg^2+^.

## 1. Introduction

*Salvia miltiorrhiza*, also named Danshen in Chinese, is a traditional medicinal herb used to treat a diversity of illnesses, especially cardiac and vascular disorders [1,2]. In the past three decades, lipid-soluble constituents of Danshen known as tanshinones were actively investigated and found to possess anti-inflammatory and immunomodulatory activities [3,4]. In the past decade, water-soluble compounds of Danshen, particularly lithospermic acid B (LSB) and its magnesium complex, have attracted more and more attention, and were found to possess several biological activities, such as attenuating atherosclerosis, improving blood circulation and treating coronary heart disease [5,6,7,8]. In fact, LSB is present as the most abundant phenolic compound in the water extract of Danshen. Therefore, the content of LSB in Danshen is regularly detected as an index for the quality control of this herb.

Similar to many natural compounds found in plant sources, LSB in Danshen is commonly identified and quantitated by high-performance liquid chromatography (HPLC) [9,10]. Although HPLC is highly sensitive and specific for the detection of LSB, it suffers from being time consuming and expensive for handling a large number of samples. In light of these disadvantages, many researchers have attempted to develop rapid tests mediated by immunochemical approaches, i.e., immunochromatographic analysis and enzyme linked immunosorbent assay (ELISA), for the specific detection of target compounds [11,12,13]. Generally, relatively tiny compounds (less than 1 kDa) lack immunogenicity and fail to elicit immune responses in animals [14,15]. In order to obtain antibodies against target compounds, isolated compounds were chemically modified and conjugated to carrier proteins to form artificial antigen complexes for animal immunization [16,17,18]. 

Artificial oil bodies (AOBs) could be stably constituted with triacylglycerols, phospholipids and integral oil-body proteins, oleosins or caleosins [19,20,21]. Recently, a system was developed to generate mono-specific antibodies against tiny molecules under the assistance of artificial oil bodies; target tiny molecules were chemically linked to recombinant caleosins and presented on the surface of AOBs for animal immunization [22]. As exemplified by biotin as a target molecule, polyclonal antibodies generated by this system specifically recognized biotin, but not the carrier proteins, which were the recombinant caleosins. It seems that this system is applicable to generate specific antibodies against relatively tiny molecules, such as medicinal drugs and natural compounds, for immunoassay. 

In this study, LSB was chemically conjugated to an engineered lysine-enriched caleosin (Cal-K). Specific antibodies against LSB (Ab-LSB) were successfully generated by immunizing hens with AOBs constituted with the LSB-conjugated caleosin. Ab-LSB harvested from eggs was used to develop an indirect competitive enzyme linked immunosorbent assay (ELISA) for the detection of LSB.

## 2. Results

### 2.1. Conjugation of LSB to Recombinant Caleosin, Cal-K

**Figure 1:**
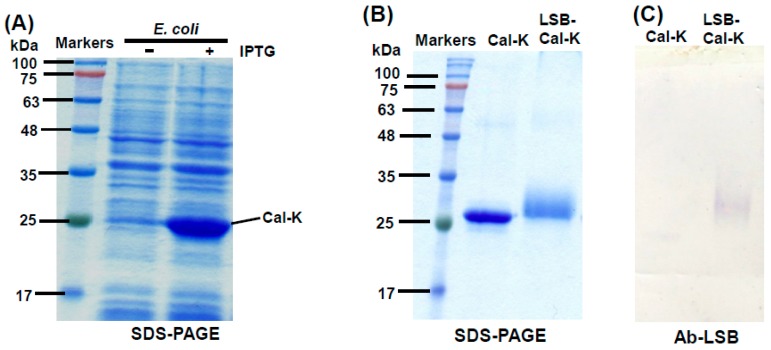
SDS-PAGE and Western blotting of the recombinant Cal-K conjugated with or without LSB. Total proteins (10 μg) of *E. coli* with the recombinant Cal-K induced by IPTG were resolved in SDS-PAGE, and molecular masses of commercial marker proteins (Genemark, Taichung, Taiwan) were indicated on the left (**A**). Purified Cal-K conjugated with or without LSB was resolved in SDS-PAGE, and molecular masses of markers were indicated (**B**). A duplicate gel of the purified Cal-K conjugated with or without LSB was transferred onto PVDF membrane and then subjected to immunoassay using Ab-LSB (**C**).

According to the protocol developed in our previous work [22], recombinant caleosin, Cal-K was over-expressed in *E. coli* cells after isopropyl β-d-thiogalactoside (IPTG) induction (Figure 1A). The Cal-K protein was eluted out from SDS-PAGE gels and used for the chemical conjugation with LSB. After the chemical conjugation, the resulting complex of LSB-conjugated caleosin (LSB-Cal-K) migrated as a smearing band with location higher than the original Cal-K putatively resulted from its increase in molecular mass with the conjugation with several molecules of LSB (Figure 1B).

### 2.2. Generation of Antibodies via AOBs Constituted with LSB-Cal-K

**Figure 2:**
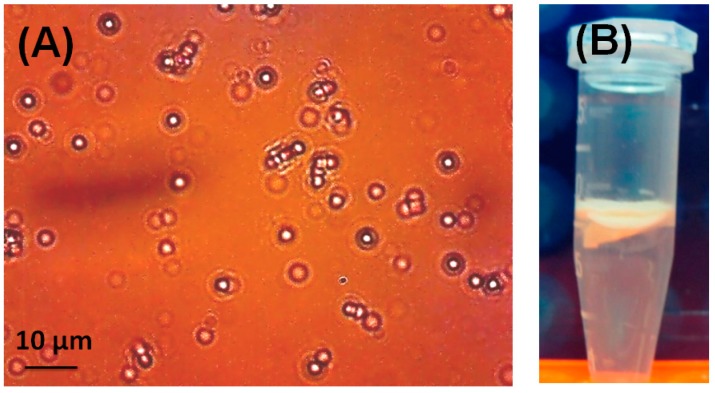
Light microscopy of artificial oil bodies (AOBs) constituted with the recombinant Cal-K conjugated with LSB (**A**). The photo was taken under 800-fold magnification. Bar represents 10 µm. The floated AOBs were packed as a milky layer on top of the solution in an Eppendorf tube after 10,000× *g* centrifugation (**B**).

LSB-Cal-K eluted from SDS-PAGE gels was subjected to the constitution of AOBs by sonication in the presence of triacylglycerols and phospholipids. After sonication, the oily triacylglycerols were encapsulated by phospholipids and LSB-Cal-K to form numerous micro-emulsions as observed in light microscopy (Figure 2A); and these micro-emulsions tended to be packed as a milky layer on the top after centrifugation (Figure 2B). Apparently, stable AOBs sheltered by LSB-Cal-K were successfully generated regardless the conjugation of several molecules of LSB on the recombinant lysine-enriched caleosin. The AOBs constituted with LSB-Cal-K were used to immunize hens, and antibodies were purified from egg yolk after immunization. Western blotting showed that the antibodies isolated from yolk seemed to specifically recognize those LSB molecules conjugated on Cal-K, but not the carrier protein, Cal-K (Figure 1C).

### 2.3. Indirect Competitive ELISA for LSB Detection

**Figure 3:**
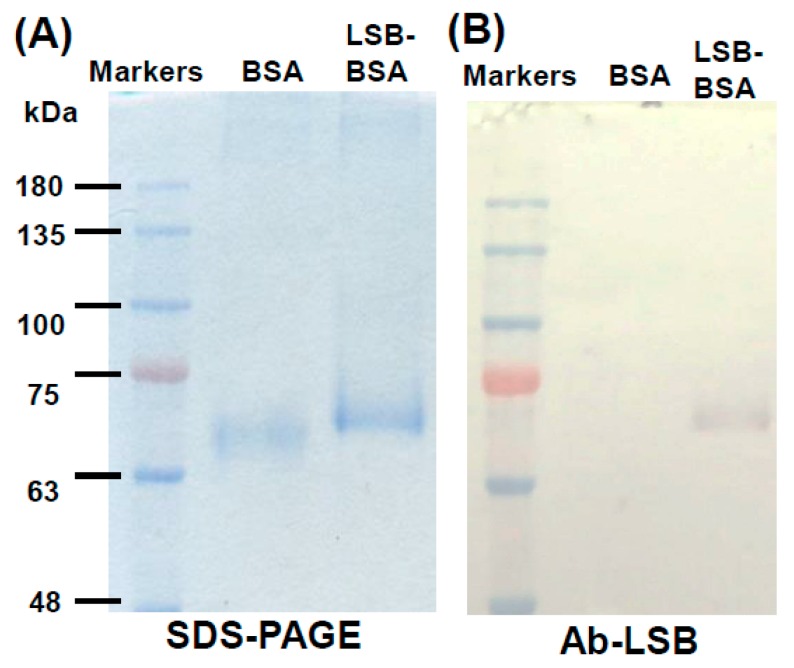
SDS-PAGE and Western blotting of BSA conjugated with or without LSB. BSA conjugated with or without LSB was resolved in SDS-PAGE (**A**), and a duplicate gel was transferred onto PVDF membrane and then subjected to immunoassay using Ab-LSB (**B**). Molecular masses of commercial marker proteins (Genemark, Taichung, Taiwan) were indicated on the left.

According to the same protocol, LSB was chemically conjugated with bovine serum albumin (BSA). Similar to the chemical conjugation of LSB and Cal-K (Figure 1B), the resulting complex of LSB-conjugated BSA (LSB-BSA) migrated to a higher position in comparison with the original BSA without chemical conjugation (Figure 3A). Again, Western blotting showed that the antibodies (Ab-LSB) seemed to specifically recognize those LSB molecules conjugated on BSA, but not the carrier protein, BSA (Figure 3B). 

**Figure 4:**
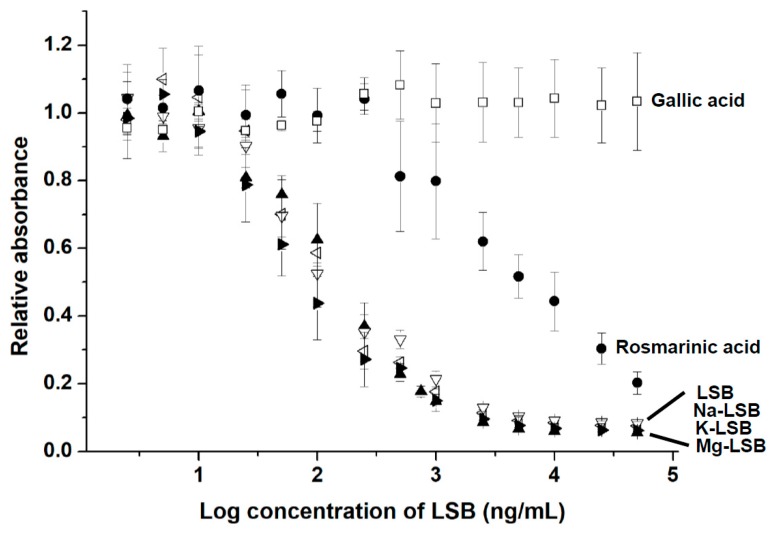
Cross-reactive curves for the detection of several phenolic compounds by indirect competitive ELISA with Ab-LSB. Each point of the curve presents the mean ± SD (replicate, *n* = 3). Na-LSB = sodium lithospermate; K-LSB = potassium lithospermate; Mg-LSB = magnesium lithoserpmate.

To detect LSB via indirect competitive ELISA, LSB-BSA was coated on a 96-well microplate. The binding between Ab-LSB and LSB-BSA on the microplate was competed dose-dependently in the presence of free LSB with a concentration ranging from 5 to 5 × 10^4^ ng/mL (Figure 4). The IC_50_ value was determined to be approximately 120 ng/mL of LSB. The limit of detection (LOD) of LSB was calculated to be 45.89 ng/mL. No significant difference was observed when LSB was complexed with a metal ion of Na^+^, K^+^ or Mg^2+^ for its competition with LSB-BSA for Ab-LSB binding. Gallic acid, a simple phenolic compound commonly found in plant sources, was unable to compete with LSB-BSA for Ab-LSB. As expected, relatively weak competition was observed for rosmarinic acid (IC_50_ ≅ 4550 ng/mL) as LSB could be structurally regarded a fusion compound covalently linked with two molecules of rosmarinic acid. 

### 2.4. Comparison of LSB Contents in Danshen Extracts Detected by HPLC and Indirect Competitive ELISA 

**Figure 5:**
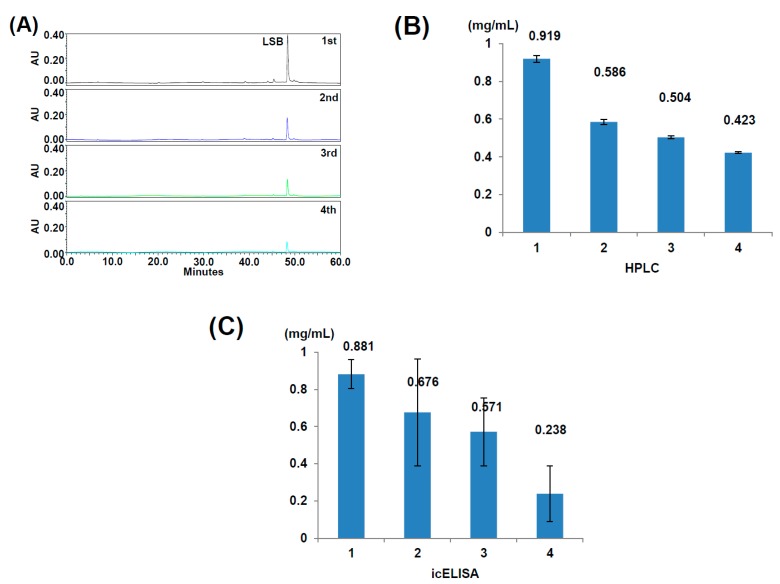
Detection of LSB contents in Danshen extracts by HPLC and indirect competitive ELISA (icELISA). Danshen of 1 g was successively extracted with 20 mL of 20% ethanol for four times, and the four extracts were analyzed by HPLC (**A**). The contents of LSB in the four extracts shown in HPLC were quantitatively measured (*n* = 3) (**B**). The four extracts were diluted by 1000 times and quantitatively measured by icELISA (*n* = 3) (**C**).

To verify the validity of detecting LSB contents in a real sample by using the indirect competitive ELISA, Danshen was successively extracted with 20% ethanol for four times. The four extracts were firstly analyzed by HPLC (Figure 5A), and the contents of LSB in the four extracts were quantitated (Figure 5B). For the detection of LSB contents with the indirect competitive ELISA, the four extracts of Danshen were diluted by 1000 times and then quantitatively measured according to the protocol developed in this study (Figure 5C). The results showed that the indirect competitive ELISA could adequately detect the contents of LSB in Danshen extracts though the variations were much higher than those detected by HPLC. 

## 3. Discussion

In this study, we successfully developed an indirect competitive ELISA method for the detection of LSB by its specific antibodies generated via the AOB system established previously [22]. Amide bonds were formed between the carboxylic acid groups of LSB and the lysine residues of the modified oil-body protein, Cal-K via the zero-length cross linking method. After the formation of AOBs, the hydrophilic LSB molecules conjugated to Cal-K were supposed to be spontaneously presented on the surface of AOBs. The surface of AOBs constituted with LSB-Cal-K seemed to be completely shielded by the conjugated LSB molecules as the carrier protein, Cal-K failed to generate antibodies (Figure 1). A similar shielding effect on the carrier protein was also observed when antibodies against biotin were generated by using the same lysine-enriched caleosin, Cal-K to present linked biotin molecules on the surface of AOBs [22]. It was demonstrated that the lysine-enriched caleosin, Cal-K is a suitable carrier for producing specific antibodies against tiny compounds via the AOB system.

Danshen is one of the major Chinese herbs imported from China to Taiwan (more than 150 tons annually), and mainly used as food additive, dietary supplement and traditional herbal medicine [23]. Without any manufacturing processes, Danshen, the original dry root of *Salvia miltiorrhiza* can be basically identified by observing its tissue section; however, the quality of this Chinese herb as well as its manufactory products requires further examination of the chemical contents. Several methods have been developed to evaluate the quality of Danshen, and the most reliable one is to examine its chemical contents extracted with water or organic solvent by HPLC/MS/MS [24,25]. This method provides precise detection for the content of each compound in the crude extract of Danshen resolved on the HPLC system. Nevertheless, the requirement for an expensive instrument, a high operating cost for each sample, and the time consuming nature of handling a large number of samples apparently restrict its application in local markets. It seems that the indirect competitive ELISA for the detection of LSB developed in this study should be a feasible tool to conveniently meet commercial utilization for a fast screening on a large number of Danshen samples. Of course, a large scale usage of this technique should be validated with larger sample sizes of Danshen prior to its commercial utilization.

As shown in Figure 4, the detection of LSB by indirect competitive ELISA may slightly affected by other phenolic compounds with structures similar to LSB, such as rosmarinic acid. Moreover, the LSB detection variation of indirect competitive ELISA is much higher than that of HPLC as shown in Figure 5. Therefore, it is unlikely that the indirect competitive ELISA is suitable to replace HPLC for the accurate quantitation of LSB. Instead, this indirect competitive ELISA is suggested to be practically used for a rough evaluation of Danshen quality in local markets to assist the decision of purchase order immediately. It is recommended that the precise content of LSB in Danshen samples should be finally determined by HPLC.

Besides hydrophilic components, mainly LSB and its related compounds, lipophilic compounds, e.g., dihydro-tanshinone, tanshinone I, tanshinone IIA, were also shown to be active ingredients that are beneficial for human health with different therapeutic effects [26]. Therefore, the contents of these tanshinones should be also detected for the quality control of Danshen. It will be interesting to see if a comparable indirect competitive ELISA method can be established for the detection of tanshinones by their specific antibodies generated via the AOB system. Whether the relatively lipophilic tanshinones linked to the Cal-K carrier are able to be presented on the surface of AOBs and immunologically reactive in animals remains to be evaluated.

## 4. Materials and Methods

### 4.1. Chemicals and Materials

1-Ethyl-3-[3-dimethylaminopropyl]carbodiimide (EDC), *N*-hydroxysulfo- succinimide (Sulfo-NHS) and 1-step PNPP solution were obtained from Thermo Scientific Inc. (Rockford, IL, USA). 1,2-Distearoyl-*sn*-glycero-3-phosphocholine, rosmarinic acid, gallic acid, sodium hydroxide, potassium hydroxide, MES monohydrate, Freund's complete adjuvant and Freund's incomplete adjuvant were purchased from Sigma (St Louis, MO, USA). Magnesium hydroxide was purchased from Showa Chemical Co. (Tokyo, Japan). Sesame oil was purchased from a local market. Lithospermic acid B (LSB) was purified and provided by the Ko Da Pharmaceutical Co. (Taoyuan, Taiwan). Sodium lithospermate (Na-LSB), potassium lithospermate (K-LSB) and magnesium lithospermate (Mg-LSB) were prepared as described previously [27]. Triton X-100 and Tris base were purchases form Amersco (Solon, OH, USA). Polyethylene glycol 8000 was purchased from Bio Basic Inc. (Markharn, Ontario, Canada). 96-well flat-bottomed Immuno Maxisorp plates were purchased from Thermo (Nunc, Roskilde, Denmark). Alkaline phosphatase conjugated affinipure Rabbit Anti-Chicken IgY was purchased from Jackson Immuno-Research (West Grove, PA, USA).

### 4.2. Over Expression and Purification of Recombinant Cal-K

The recombinant caleosin with 17 extra lysine residues was selected for this study and over-expressed in *E. coli* cells after IPTG induction according to the protocol developed in our previous work [22]. This lysine-enriched caleosin (Cal-K) was resolved on SDS-PAGE of 12.5% acylamide and stained with Coomassie blue R-250 after the electrophoresis. The stained Cal-K was eluted out from gels and precipitated by adding equal volume of pre-cooled acetone. The protein solution was placed at −20 °C for 1 h and centrifuged at 10,000× *g* for 20 min. After centrifugation, Cal-K pellet was dissolved in the phosphate buffered saline (PBS) containing 3% Triton and used in the following conjugation reaction with LSB. 

### 4.3. Conjugation of LSB to Cal-K and BSA

In an Eppendorf tube, 120 μL of 10 mM LSB was added with 100 μL of 10 mM EDC, 100 μL of 10 mM sulfo-NHS solution, and 45 μL of 200 mM MES buffer, pH 6. The mixture solution was then added with 90 μL of Cal-K solution or 50 μL of BSA solution (3.75 mg BSA in 1 mL of PBS buffer) to initiate the conjugation between LSB and Cal-K or BSA. After reaction at 60 °C in a dry bath for 2 h, 30 μL of 40 mM hydroxyamine solution was added to quench the reaction. The resulting product, LSB-Cal-K was collected and mixed with 15 times volume of the PBS buffer in a 50-mL centrifuge tube and centrifuged at 10,000× *g* at 4 °C for 20 min. After centrifugation, the insoluble LSB-Cal-K pellet was re-suspended in the PBS buffer and its protein concentration was determined by the BCA Protein Assay kit (Pierce). The resulting product, LSB-BSA was dialyzed against 500 mL of the PBS buffer at 4 °C for 3 times. Finally, LSB-BSA was frozen, lyophilized and dissolved in distilled water to a final concentration of approximately 275 μg/mL.

### 4.4. Preparation of AOBs for Antibody Generation

LSB-Cal-K was used to constitute AOBs according to the protocol developed previously [28]. Briefly, 150 μg of 1,2-Distearoyl-*sn*-glycero-3-phosphocholine (DSPC) dissolved in chloroform was set at the bottom of an Eppendorf tube, and the solvent was evaporated in a chemical hood overnight. Then, 20 mg of sesame oil, 250 μg of LSB-Cal-K and 750 μL of PBS buffer were added into the DSPC-coated tube prior to the sonication. To generate antibodies, 750 μL of AOBs constituted with LSB-Cal-K were mixed with equal volume of Freund's complete adjuvant and injected into two hens. Hens received two subsequent booster injections with Freund's incomplete adjuvant at 2-week intervals. Eggs were collected from day 0 until the end of the experiment and stored at 4 °C. Following our previous study [22], the protocols and care administered to the animals were approved by the Institutional Animal Care and Use Committee of the National Chung−Hsing University with the approval number of IACUC 99-52: Development of biotechnological products based on artificial oil bodies.

### 4.5. Purification of Antibodies from Egg Yolk

Antibodies (IgY) were extracted from egg yolks by following the modified method of Polson et al. using polyethylene glycol (PEG) and ethanol precipitation [29,30]. The egg yolk was separated from egg white, rinsed the white out of the membrane surface with distilled water, and rolled on moisturized tissue paper to remove adhering proteins. Then, the yolk membrane was punctured and the yolk was collected into a 50-mL centrifuge tube. The phosphate buffer (0.1 M phosphate, pH 7.6 with 0.01% sodium azide) containing 4.5% PEG 8000 (*w*/*v*) was added and mixed with the yolk to a final concentration of 3.5%. The mixture was centrifuged at 4 °C at 5,000× *g* for 20 min on Kubota 5400R. After centrifugation, the supernatant was filtered through Whatman #1 paper, and then added with PEG 8000 powder to a final concentration of 12%. The mixture was subjected to centrifugation again, and the pellet were collected and dissolved in 35 mL of 12% PEG 8000 in the phosphate buffer. After one more centrifugation, the precipitate was dissolved in 3 mL of the phosphate buffer, mixed equal volume of −20 °C pre-cooled 50% ethanol (*v*/*v*), and kept at −20 °C for 30 min. This mixture was centrifuged at −10 °C, 10,000× *g* for 25 min. Finally, the precipitate was dissolved in 3 mL of the phosphate buffer and stored at −20 °C. The concentration of antibodies was roughly 15 mg/mL, and the Ab-LSB could be diluted by 1000–3000 times for Western blotting.

### 4.6. Western Blotting

Cal-K and BSA with or without conjugation with LSB were resolved on SDS-PAGE with a 12.5%, 29:1 (acrylamide/bisacrylamide) gel. After electrophoresis, the SDS-PAGE gels were transferred onto PVDF membrane (Millipore) in the Bio-Rad Trans-Blot system. Sequentially, the membrane was blocked with 5% skimmed milk (*w*/*v*) in TBST buffer (20 mM Tris base, 500 mM NaCl, 0.05% Tween-20, pH 7.5) at room temperature for 1 h, and rinsed three times with TBST before adding primary antibody solution, Ab-LSB (dilution ratio, 1:2000) purified from egg yolk. The primary immuno-detecting process was reacted at room temperature for 1 h. After rinsed with TBST for 3 times, secondary antibody solution (dilution ratio, 1:5000), alkaline phosphatase conjugated rabbit anti-Chicken IgY, was incubated with the membrane at room temperature for 30 min. Finally, the color was developed by incubating membrane with BCIP/NBT solution (Promega). 

### 4.7. Indirect Competitive ELISA

For coating, 100 μL of LSB-BSA (2 μg/mL) was added into a 96-well microplate and shaken at 4 °C overnight. After coating, 200 μL of 5% skimmed milk in the PBS buffer with 0.05% Tween-20 (PBST) was loaded into each well for 2 h, and then washed with 200 μL of PBST for three times. For competition with the binding between LSB-BSA and Ab-LSB purified from yolk (1:500 dilution; 75 μL/well), LSB and its metal complexes of different concentrations ranging from 5 to 5 × 10^4^ ng/mL were added (75 μL/well) to the microplate. The competition was kept for 1 h at 37 °C, and then rinsed with PBST buffer for three times. Sequentially, 150 μL of secondary antibody, alkaline phosphatase conjugated rabbit anti-Chicken IgY solution (1:2500 dilution) was added and incubated for 1 h at 37 °C. After being rinsed three times with PBST buffer, 100 μL of 1-step PNPP solution was poured into the microplate for 30 min, and then quenched by adding 50 μL of 2N NaOH. Finally, the absorbance at 405 nm was detected by TECAN infinite M200 Pro. The Limit of Blank (LOB) was estimated by measuring replicates of a blank sample and calculating the mean result and the standard deviation (SD).

LOB = mean _blank_ + 1.645 × (SD_blank_) (1)

After calculating LOB, the limit of detection (LOD) was calculated according to LOD = LOB + 1.645 (SD _low concentration sample_). Here, the low concentration of LSB was 2.5 ng/mL. 

The standard curve was fitted using the following equation:

Y = A + (B − A)/[1 + (X/C)^D^] (2)

where A and B are the values at minimum and maximum relative absorbance of the curve, respectively; C is the concentration of the analyte resulting in 50% inhibition (IC_50_) and D is the curve slope at the inflection point.

Four parameter logistic (4PL) curve of LSB was plotted with a minimum of 0.05252, maximum of 1.0262, inflection point of 2.0758, and hill coefficient of −1.0853.

### 4.8. Detection of LSB Contents in Danshen Extracts by HPLC and the Indirect Competitive ELISA

Danshen of 1 g was extracted with 20 mL of 20% ethanol at room temperature for 3 h. The extraction process was repeated for four times, and the four successive extracts were filtered through filter paper (Advantec No.1) and collected separately for the following analyses. The four extracts were filtered through 0.45 μm filter and analyzed by HPLC coupled to a Waters Corp. 600 controller pump with 2996 photodiode array detector and 717 autosampler (Milford, MA, USA). The separation was achieved using a Mightysil RP-18 GP column (250 × 4.6 mm i.d., 5 μm) from Kanto Chemical Co. (Tokyo, Japan). The HPLC mobile phase comprised (A) 100% acetonitrile with 0.2% formic acid and (B) 0.2% formic acid. The program for gradient elution comprised 12% solvent A and 88% solvent B for 0–5 min, 20% solvent A and 80% solvent B for 5–20 min, 23% solvent A and 77% solvent B for 20–30 min, 30% solvent A and 70% solvent B for 30–40 min, and 12% solvent A and 88% solvent B for 40–45 min. The detection wavelength was set at 286 nm. The LSB contents of the four extracts in the HPLC analysis were quantitated by using the linear standard regression equations. To compare the detection of LSB contents with the indirect competitive ELISA with the above HPLC analysis, the four extracts of Danshen were diluted by 1000 times and then quantitatively measured according to the method described in the previous section. Data were expressed as means ± SD of three replicates.
